# Systematic Review and Meta-Analysis of Perioperative Intravenous Tranexamic Acid Use in Spinal Surgery

**DOI:** 10.1371/journal.pone.0055436

**Published:** 2013-02-12

**Authors:** Baohui Yang, Haopeng Li, Dong Wang, Xijing He, Chun Zhang, Pinglin Yang

**Affiliations:** Department of Orthopaedic Surgery, The 2nd Affiliated Hospital of Medical College, Xi'an Jiaotong University, Xi'an, Shaanxi Province, People’s Republic of China; Università Vita-Salute San Raffaele, Italy

## Abstract

**Background:**

Tranexamic acid (TXA) is well-established as a versatile oral, intramuscular, and intravenous (IV) antifibrinolytic agent. However, the efficacy of IV TXA in reducing perioperative blood transfusion in spinal surgery is poorly documented.

**Methodology:**

We conducted a meta-analysis of randomized controlled trials (RCTs) and quasi-randomized (qi-RCTs) trials that included patients for various spinal surgeries, such as adolescent scoliosis surgery administered with perioperative IV TXA according to Cochrane Collaboration guidelines using electronic PubMed, Cochrane Central Register of Controlled Trials, and Embase databases. Additional journal articles and conference proceedings were manually located by two independent researchers.

**Results:**

Totally, nine studies were included, with a total sample size of 581 patients. Mean blood loss was decreased in patients treated with perioperative IV TXA by 128.28 ml intraoperatively (ranging from 33.84 to 222.73 ml), 98.49 ml postoperatively (ranging from 83.22 to 113.77 ml), and 389.21 ml combined (ranging from 177.83 to 600.60 ml). The mean volume of transfused packed cells were reduced by 134.55 ml (ranging 51.64 to 217.46) (95% CI; P = 0.0001). Overall, the number of patients treated with TXA who required blood transfusions was lower by 35% than that of patients treated with the comparator and who required blood transfusions (RR 0.65; 95% CI; 0.53 to 0.85; P<0.0001, I^2^ = 0%). A dose-independent beneficial effect of TXA was observed, and confirmed in subgroup and sensitivity analyses. A total of seven studies reported DVT data. The study containing only a single DVT case was not combined.

**Conclusions:**

The blood loss was reduced in spinal surgery patients with perioperative IV TXA treatment. Also the percentage of spinal surgery patients who required blood transfusion was significantly decreased. Further evaluation is required to confirm our findings before TXA can be safely used in patients undergoing spine surgery.

## Introduction

Server perioperative blood loss during procedures is a major concern during procedures, such as spinal surgery; therefore, allogeneic blood transfusion is a common requirement [1]. However, it may present additional risks to patients that might and result in longer duration of hospitalization and impair patient outcomes, such as adverse immunological reactions, disease transmission, intravascular haemolysis, transfusion induced coagulopathy, renal impairment or failure, and even increased mortality [2], [3]. Significant perioperative bleeding during spinal surgery is also a unique risk for spinal epidural hematoma formation, which may potentially result in the development of spinal cord or cauda equina compression [4], [5]. Thus, it is very important to control perioperative bleeding for maintaining hemodynamic equilibrium and decreasing the risk of postoperative epidural hematoma.

A variety of contemporary blood-conservation techniques have been used to reduce exposure to allogeneic blood, including controlled hypotension, regional anesthesia, autologous blood transfusion, intraoperative blood salvage, and administration of various intravenous (IV), intramuscular, and oral medications [3]. However, these hemostatic techniques are not applied in spinal surgeries consistently. Tranexamic acid (TXA), a synthetic antifibrinolytic agent, is one common medication that could competitively block the lysine-binding sites of plasminogen, plasmin, and the tissue plasminogen activator, thereby retard fibrinolysis and blood clot degradation [6]. Antifibrinolytics have been demonstrated to reduce blood transfusion requirement during cardiac surgery, total knee arthroplasty, and urology surgery [7], [8], [9], suggesting that TXA may have a similar effect in spinal surgery.

Based on a recent comprehensive evaluation and meta-analysis of the effects of antifibrinolytic drug on both bleeding and transfusion, it could significantly reduce blood loss, thus reduce the need for allogeneic red blood cell transfusions in surgical patients [10]. To date, no study has specifically examined these effects on spinal surgery patients, despite the prevalence of clinical implementation of these treatments.

Fibrinolysis represents a unique challenge in spinal surgery and postsurgical care. It has been suggested that antifibrinolytic drugs, such as perioperative IV TXA, should be applied more routinely in clinical spinal surgeries. Therefore, we conducted a systematic review and meta-analysis of the efficacy of TXA in reducing blood loss and exposure to perioperative blood transfusion during spinal. We also further examined the association of IV TXA with complication rates.

## Materials and Methods

### Study Design

A meta-analysis and systematic review was conducted according to a predefined guidelines provided by the Cochrane Collaboration (2008) [11]. All data were reported according to the Quality of Reporting for Meta-analyses provided by the Handbook for Systematic Reviews of Interventions Version 5.0.0 [12].

### Study Selection

We performed a comprehensive search based on multi-database electronic and manual literature searches to identify relevant controlled trials (RCTs) and quasi-randomized trials (qi-RCTs) published between December 1966 and September 2012 involving patients undergoing a variety of spinal surgery, including adolescent spinal scoliosis surgery and other surgery types. Electronic searches were conducted in database including the PubMed, Cochrane Central Register of Controlled Trials, and Embase databases without language restrictions. The search terms included “antifibrinolytics”, “tranexamic acid”, “cyklokapron”, “aprotinin”, “trasylol”, “epsilon aminocaproic acid”, “amicar” and “randomized controlled trials”. Manual searches were conducted only in English using the Orthopedics China Biological Medicine database.

The studies were included if they met the following criteria: (i) participants underwent spinal surgery; (ii) interventions included administration of perioperative IV TXA; (iii) results of a placebo (control) group were reported; (iv) reported outcomes including intraoperative, postoperative, and total blood loss (primary outcomes); and (v) reported outcomes included the number of patients receiving allogeneic blood transfusion, transfusion packed cells volumes, and deep venous thrombosis (DVT) incidence (secondary outcomes).

Studies reporting the results of oral or intramuscular TXA interventions were excluded. Non-randomized studies and studies reporting the results of spinal revision were also excluded. In this study, the allocation sequence of RCTs was considered to be unpredictable (e.g. selection by random computer generated sequences), while that of qi-RCTs was considered to be predictable (e.g. selection by medical record numbers or birth dates).

### Data Collection and Analysis

The initial electronic databases searches to identify potential studies for inclusion based on title and abstract information were performed by two independent authors (BaoHui Yang and HaoPeng Li). Complete study reports were assessed for inclusion independently by both authors. In cases of insufficient data, authors were contacted for information and clarification. Final determination of inclusion was made by the senior author (Xijing He). If any author presented an objection to inclusion because studies do not meet the inclusion criteria, the study was excluded from the analysis. References and data for each included study were carefully cross-checked to ensure no overlapping data was presented, thereby to ensure the integrity of the meta-analysis results.

### Data Extraction and Management

The general characteristics, treatment or intervention types, and outcomes were recorded for each study. General characteristics included study design, publication date, participant demographics and number, geographic location, and interventions. For all included studies, treatment and interventions were recorded, including IV TXA dose regimen and timing, anesthesia method(s), and surgical intervention(s). Measured outcomes included the primary and secondary outcomes as described previously. In addition, all adverse outcomes were recorded. For studies without adverse outcomes, author(s) were contacted via e-mail for confirmation or more information regarding adverse events, if necessary.

### Bias Assessment

The included studies were assessed for risk of bias by 2 independent researchers and a managing reviewer according to the Cochrane guidelines [11]. Individual methodological domain reporting (randomization sequence, allocation concealment, and blinding) for included study was graded accordingly: (i) adequate = methods were reported and appropriate; (ii) inadequate = methods were reported but inappropriate; or (iii) unclear = methods were not reported. A quality scale or checklist was not employed in order to reduce inherent inaccuracy due to scale validity in accordance with Cochrane guidelines [12].

### Meta-analysis Assessment of Treatment Effects and Outcomes

Meta-analysis on the data, including effects and outcomes of treatments, was performed using the Mantel-Haenszel method with Review Manager software (RevMan version 5.0, Cochrane Collaboration, Germany). For continuous data, such as blood loss, mean ± standard deviation (mean ± SD) was used to calculate the weighted mean difference (WMD) and 95% confidence interval (CI). For Dichotomous data, relative risk (RR) and 95% CI were applied.

### Assessment of Heterogeneity

Statistical heterogeneity was assessed using the value of I^2^ and the result of the chi-squared test A p-value <0.1 and an I^2^ value >50% were considered suggestive of statistical heterogeneity, prompting a random effects modeling estimate. Otherwise, a fixed effects approach was used. However, a non-significant chi-squared test result (a p-value ≥0.1 and an I^2^ value ≤50%) only indicated a lack of evidence for heterogeneity, but not implied necessarily homogeneity, as there may have been insufficient power to be able to detect heterogeneity.

### Subgroup Analysis and Investigation of Heterogeneity

If any heterogeneity was observed, the causes of heterogeneity was first analyzed and then subjected to sub-group treatment. If the statistical heterogeneity cannot be eliminated, a random effect model was used for the combined analysis of the studies to examine the clinical consistency.

## Results

### Inclusion of Studies

**Figure 1:**
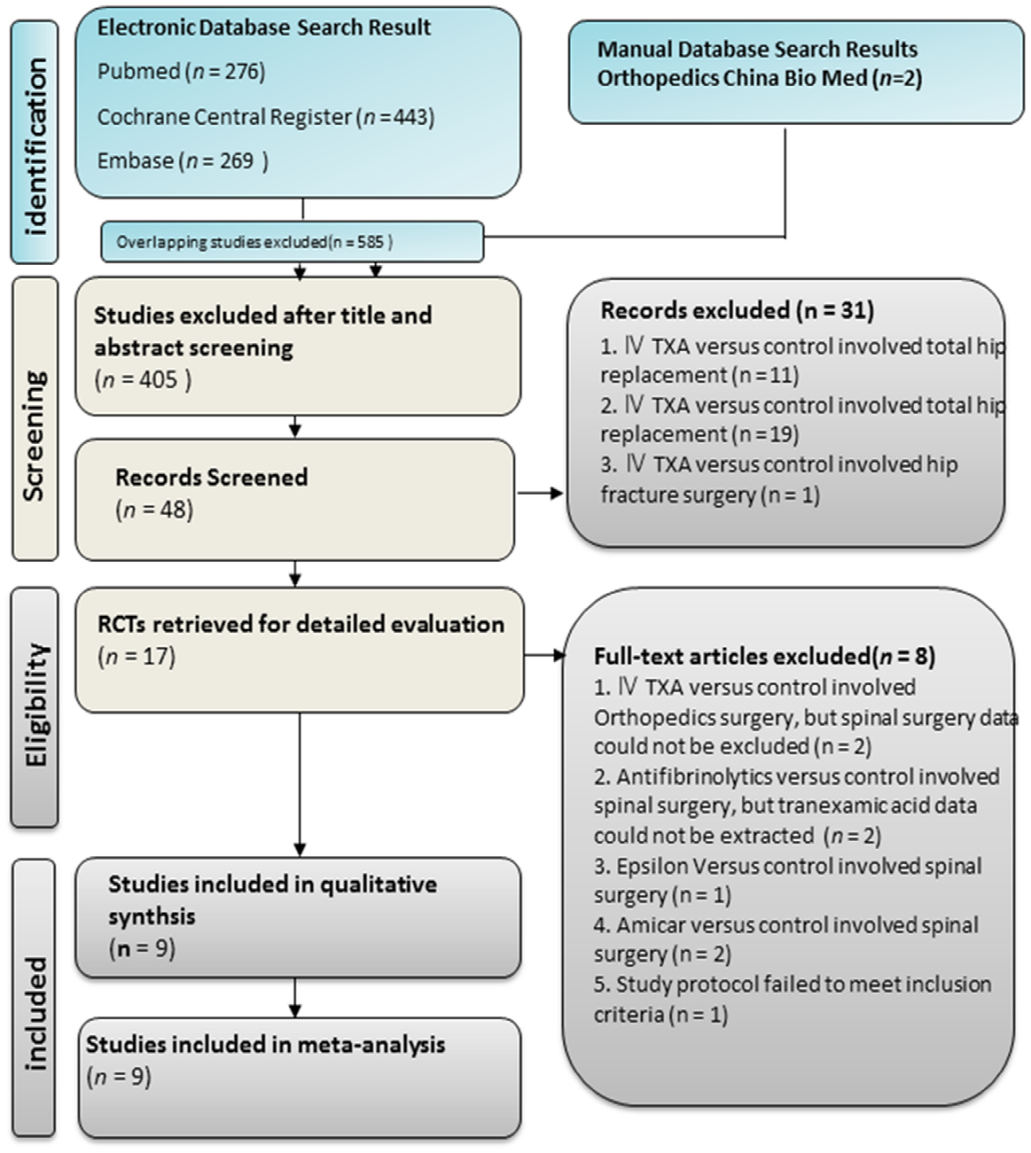
Method for study search and selection for inclusion.

The initial search identified a total of 990 articles from the electronic database (Pubmed: n = 276; Cochrane: n = 443; Embase: n = 269) and manual search (Orthopedics China Bio Med: n = 2). After exclusion of 585 studies containing overlapping data or appearing in more than one database, 445 studies remained. After screening the titles and abstracts, 397 studies that do not meet the inclusion criteria were further excluded, and 31 additional studies without specific information in spinal surgery also were excluded. In the remained 17 studies, 8 studies were excluded based on the exclusion criteria provided. Finally, 9 studies (5 RCTs and 2 qi-RCTs) that met all inclusion criteria were included in our study, including 7 studies published in English, 1 study in Chinese, and 1 study in Korean. These 9 studies included a total of 581 patients. A detailed flow chat for selection is shown in **Figure 1**.

### Quality Assessment for Included Studies

**Figure 2:**
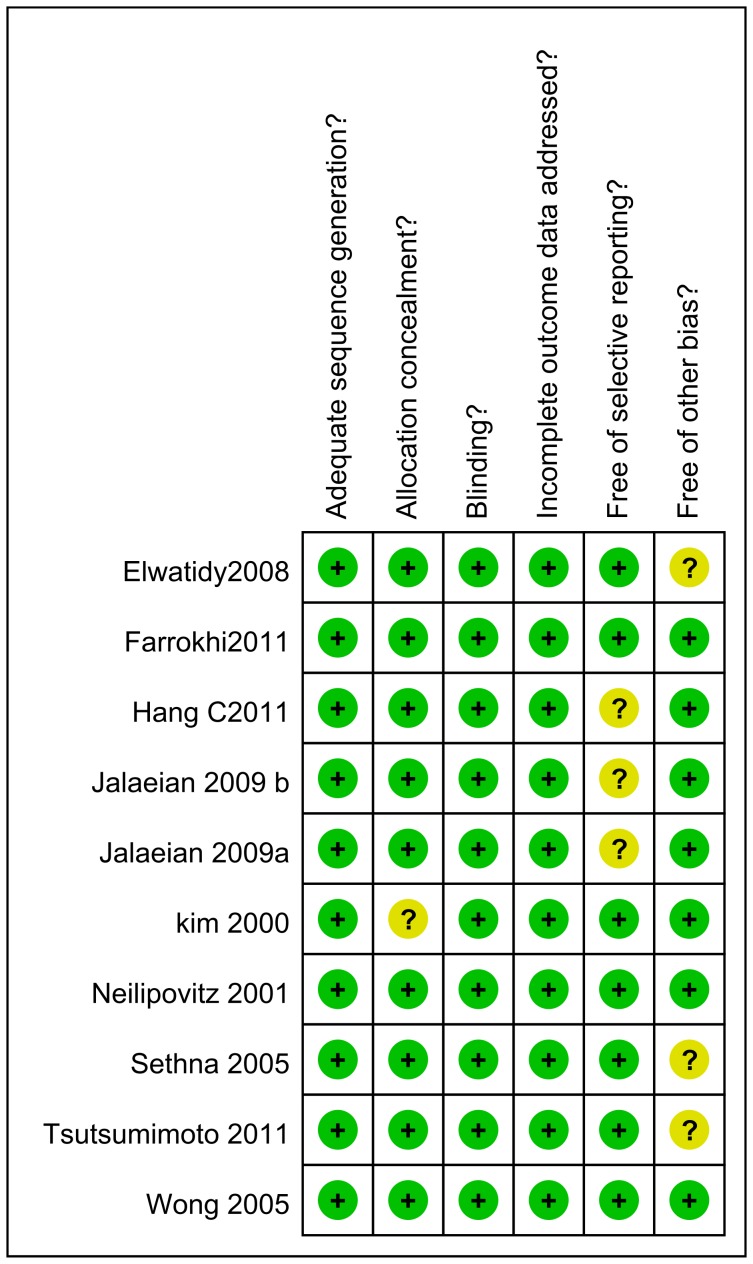
Risk of bias summary. review authors' judgments about each risk of bias item for each included study. + is “yes”, − is “no”, ? is “unclear”.

For all included studies, the methodological quality represented a minimal risk of bias-based error or uncertainty. All studies provided detailed information on the randomization techniques applied, including randomization generated by computer programs [13], [14], [15], [16], [17] or manual random number selection [18]. Except 2 quasi-randomized studies by Elwatidy et al. [19] and Tsutsumimoto et al [20] using an odd and even numbering system, all the other 7 studies were fully randomized. Allocation concealment was unclear in one study [18]. 6 studies applied double-blind techniques [13], [14], [15], [18], [19], [21], and 3 study applied the single-blind study design [16], [18], [20], indicating a potential selection bias. A summary of the methodological domain assessments for each included study is shown in **Figure 2**.

### Study Characteristics

As shown in **Table S1 in Supporting Information S1**, 9 studies in our meta-analysis included 2 studies of scoliosis [14], [21], 4 studies of posterior spinal canal decompression and internal fixation [13], [14], [16], [18], 1 study of spinal fixation [15], 1 study of lumbar hernial disc resection [21], and 1 study of “French-door” cervical laminoplasty [20]. All studies focused on the posterior, except one study with a focus on both anterior and posterior [14]. No significant differences in the preoperative baseline information were observed between the experimental and placebo groups, including consistent preoperative hemoglobin, age, gender, height, weight, and American Society of Anesthesiologists (ASA) grading. In the case of scoliosis surgeries, no significant difference in fusion ranges were observed between experimental and placebo groups. In all studies, intraoperative general anesthesia was applied, and a placebo (normal saline) was consistently administered.

For each study, TXA was administered by IV, though variant dosages (ranging form 10–100 mg/kg) and delivery timings were applied. In 2 studies, a single IV bolus was administered preoperatively [18], [20]. Administration of a prolonged infusion of 1 g every two hours was used in 2 studies [16], [21]. In the 5 other studies, boluses were repeatedly administered [13], [14], [15], [17], [19].

DVT screening was conducted by either clinical assessment [14], [15], [16], [17], [20] or routine ultrasound examination [13], [18]. In 5 studies, a transfusion trigger was initiated [13], [14], [16], [19], [21], resulting in decreased hemoglobin or hematocrit levels.

### Effects on Blood Loss and Transfusion Rates

**Figure 3:**
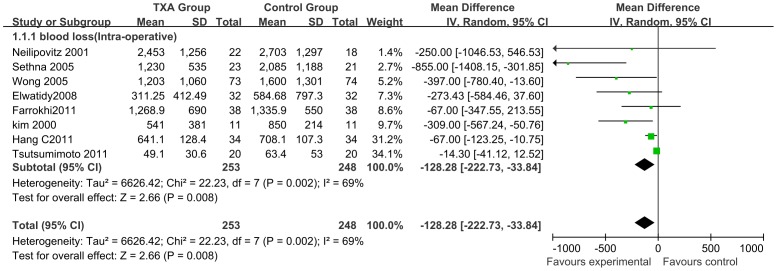
Forest plot diagram showing the effect of TXA on intra-operative blood loss. The black diamond signifies that the mean difference is in favour of TXA. The size of each square depends on the weight of each study. A green square is given to continuous outcomes.

A total of 8 studies (n = 501; 253 experimental and 248 control patients) [13]–[20] reported detailed data on intraoperative blood loss. Perioperative IV TXA administration was shown to significantly reduce intraoperative blood loss by a mean volume of 128.28 ml, ranging from 33.84 to 222.73 ml compared with that in control patients (95% CI; P = 0.008; I^2^ = 69%) (**Fig. 3**).

**Figure 4:**
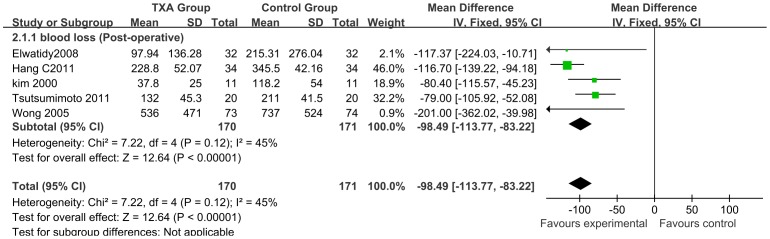
Forest plot diagram showing the effect of TXA on post-operative blood loss. The black diamond signifies that the mean difference is in favour of TXA. The size of each square depends on the weight of each study. A green square is given to continuous outcomes.

A total of 5 studies (n = 341; 170 experimental and 171 control patients) [13], [16]–[18], [20] provided data on postoperative blood loss outcomes. Perioperative IV TXA administration significantly reduced postoperative blood loss by a mean volume of 98.49 ml, ranging from 83.22 to 113.77 compared with that in control patients (95% CI; P<0.00001; I^2^ = 45%) (**Fig. 4**).

**Figure 5:**
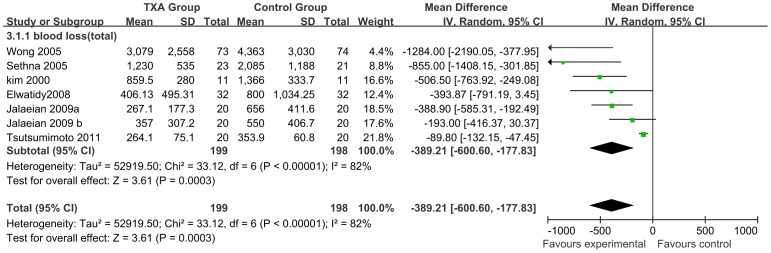
Forest plot diagram showing the effect of TXA on total blood loss. The black diamond signifies that the mean difference is in favour of TXA. The size of each square depends on the weight of each study. A green square is given to continuous outcomes.

A total of 6 studies (n = 397; 199 experimental and 198 control patients) [13], [16], [17]–[19], [21] reported total blood loss, and a minimal heterogeneity was observed among these studies (I^2^ = 82%). Using the random-effects model, perioperative IV TXA was demonstrated to significantly reduce total blood loss by a mean of 389.21 ml, ranging from 177.83 to 600.60 than that in control patients (95% CI; P = 0.0003) (**Fig. 5**).

**Figure 6:**
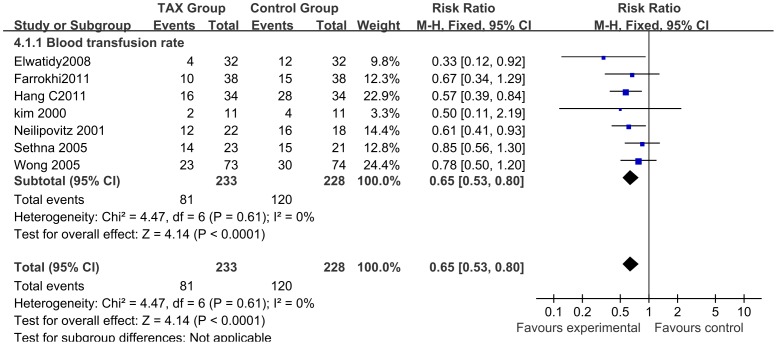
Forest plot diagram showing the effect of TXA on blood transfusion rate. The black diamond signifies that the mean difference is in favour of TXA. The size of each square depends on the weight of each study. A blue square is given to dichotomous outcomes.

A total of 7 studies (n = 461; 233 experimental and 228 control patients) [13]–[17], [19], [20] contributed data on blood transfusion occurrence rates following spinal surgery. Perioperative IV TXA administration reduced the rate of allogeneic blood transfusion by a relative 35% RR 0.65; 95% CI; 0.53 to 0.80; P<0.0001, I^2^ = 0%) (**Fig. 6**).

**Figure 7:**
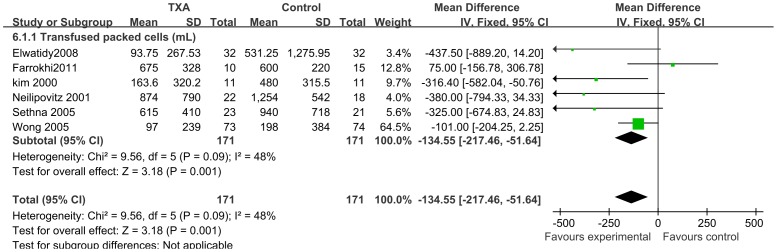
Forest plot diagram showing the effect of TXA on Amount of transfusion.

A total of 6 studies (n = 342; 171 experimental and 171 control patients) [13], [14], [15], [18], [19], [21] contributed data on transfused packed cells volume outcomes. Perioperative IV TXA administration significantly reduced transfused packed cells volumes by a mean of 134.55 ml, ranging from 51.64 to 217.46 than in control patients (95% CI; P<0.0001) (**Fig. 7**).

**Figure 8:**
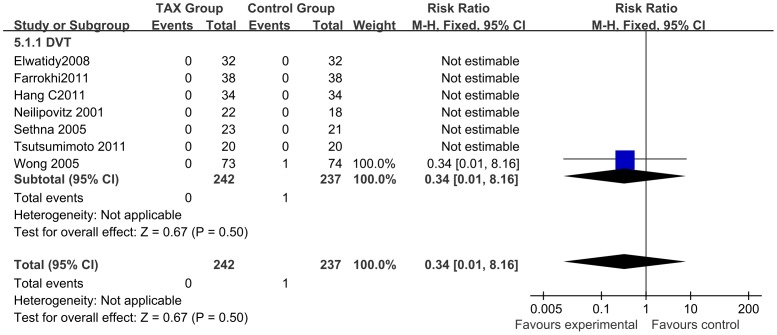
Forest plot diagram showing the effect of TXA on DVT.

A total of 7 studies (n = 479) reported DVT data. One study with only a single DVT case was not included [13] (**Fig. 8**). Additionally, all the reported adverse events, including hematoma formation and various complications due to infection, were recorded without merge due to lack of consistent and comparable data. Notably, all studies clearly states that no statistically significant difference in other adverse events existed between the experimental and control (placebo) groups.

### Subgroup Analysis and Investigation of Heterogeneity

Heterogeneity was first analyzed and then subjected to subgroup treatment. Based on heterogeneity data, subgroup analysis was designed for anesthesia type, IV TXA dose regime and timing, spinal surgery delivery, transfusion triggers, and surgical methods for studies containing sufficient and complete data sets for each of these variables. However, considering of the incomplete data, the included studies were finally divided by TXA loading drug dose as low (≤15 mg/kg) and high (>15 mg/kg) doses. Subgroup analysis revealed that both high [17], [19] and low [13]–[16], [18] doses of periop`erative IV TXA can reduce intraoperative, postoperative, and total blood loss. (Table S2 and Table S3 **in Supporting Information S1**).

Sensitivity analysis showed that transfusion rates in patients treated with perioperative IV TXA were lower than that in control patients (RR 0.53; 95% CI 0.34 to 0.85; P = 0.008) (**Table S4 in Supporting Information S1**). 2 quasi-randomized control trials [19], [20] were excluded from this analysis, and the results before and after the exclusion were consistent, demonstrating the reliability of these results.

**Figure 9:**
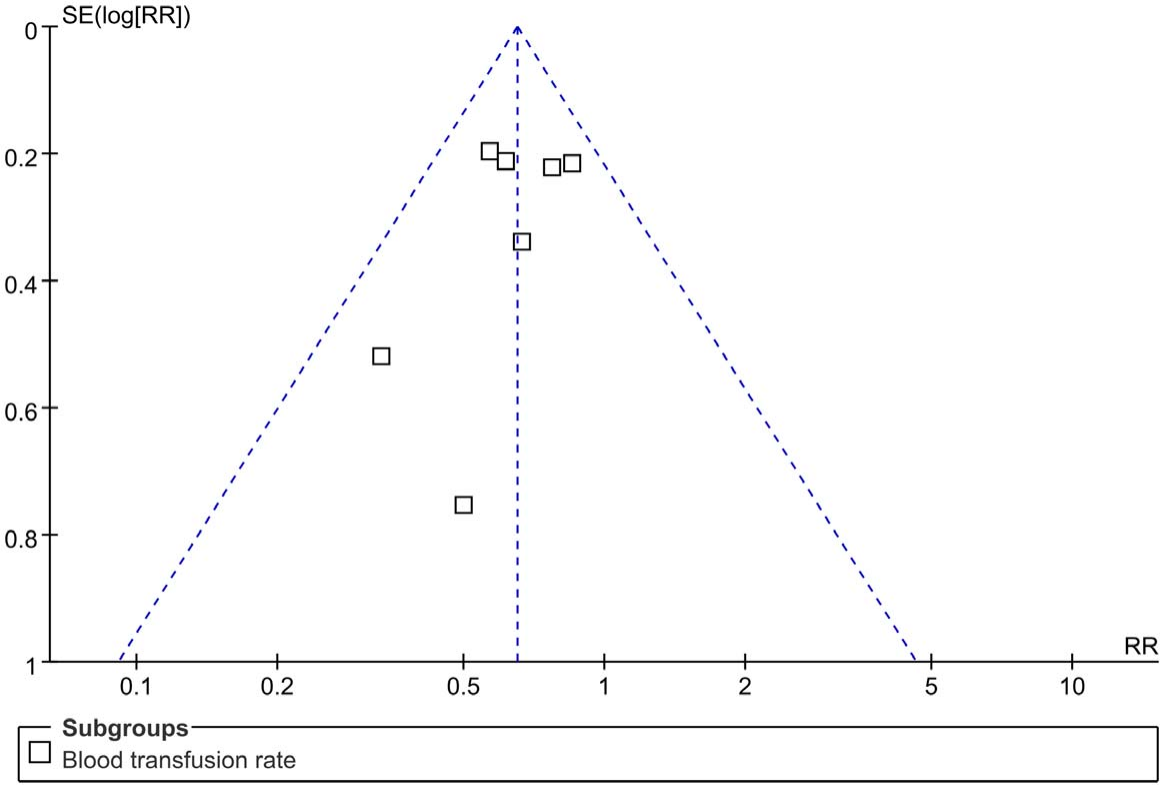
Funnel plot to assess publication. Funnel plot to assess publication for the most frequently reported outcome –blood transfusion rate.

Blood transfusion rates were also used to generate the funnel plot analysis of publication bias (**Fig. 9**). The asymmetric characteristic of the resultant plot indicated the presence of publication bias.

## Discussion

Blood loss both during and after spinal surgery, a major concern, makes it necessary to perform transfusion, which may present more risks to patients. Thus, a better understanding of the clinical effects of hemostatic techniques is required. The synthetic antifibrinolytic agent, TXA, has been demonstrated to reduce the need for blood transfusions, thereby prevent adverse complications, such as transfusion reactions and spinal epidural hematoma formation. Evaluation on the dose- and time-related efficacy, as well as a widespread implementation of current hemostatic techniques may reduce the need for transfusion procedures in clinical spinal surgery patients and improve overall patient outcomes.

Transfusion is common in patients undergoing spinal surgery. Extensive evidence has demonstrated that patients undergoing such surgery and receiving transfusion are at risk of numerous moderate and severe complications. Thus, a safe and effective intervention to reduce the number of blood units to these patients during and immediately following surgery is required. TXA has been successfully applied to treat major hemorrhaging in adult spinal surgery [22]. The efficacy and safety of the intravenous TXA, and epsilon-aminocaproic acid were further evaluated in a meta-analysis of 23 trials with 1,268 participants by Zufferey et al., demonstrating that these agents could significantly reduce the risk of allogeneic erythrocyte transfusion during orthopedic surgeries [23]. A large body of literature has accumulated to confirm the effectiveness of these agents [13]–[15], [19], [20], [21], [22], although the studies have questioned the effectiveness of managing intraoperative blood loss with TXA [15]. Although TXA and aprotinin are both effective, TXA has been regarded as a better choice as it clinically available [25]. Thus, whether TXA is the optimal solution for wide implementation to reduce blood transfusion in spinal surgery remains a topic of controversy.

TXA has been demonstrated to be effective in numerous spinal surgeries in adults, adolescents, and children, suggesting that it might be a potential treatment for various surgical procedures. In scoliosis correction surgery, TXA was shown to be effective when used along with other blood conservation measures [23]. Consistently, the similar effects were observed in adolescent idiopathic scoliosis surgeries both in adult and adolescent [24]. Furthermore, higher postoperative hemoglobin concentrations and decreased blood loss were observed in posterior lumbar surgery [25] and pediatric vertebral column resection procedures [26] when TXA were administrated. Although TXA has been widely applied in various surgical procedures, the techniques for assessing plasma and blood TXA levels have only been developed recently [27]. Further investigations on effects in different types of spinal surgery are required to achieve a better regimen with optimal dosage and administration timing.

A systematic review and meta-analysis [28] reported the effectiveness of antifibrinolytic agents in reducing blood loss and transfusion rates in various spinal surgery patients, suggesting that such agents should be considered by surgeons and anesthesiologists in perioperative administration. However, there are several limitations in that study, including the use of dated studies, and a mixture of study types that may result in inconclusive findings when subjected to rigorous statistical review. In our presented meta-analysis review, only RCTs and qi-RCTs were included, thus to improve the methodological quality and minimize bias-based errors. Notably, our finding confirms that when patients were treated TXA perioperatively, the blood loss was reduced. Furthermore, the number of patients who required allogeneic blood transfusions was lower by 35%. This persistent positive effect of IV TXA was dose- and administration timing-independent.

In order to fully investigate the clinical effects associated with TXA administration, the included studies were selected independently of whether they supported the TXA use, including 2 studies that did not support the routine use of TXA in spinal surgery. In a double-blind RCT of 76 patients in spinal fixation surgery, TXA was administered upon induction of anesthesia for 10 min, followed by IV infusion of TXA at a rate of 1 mg/kg/h in 38 patients, and an equivalent protocol of normal saline in 38 patients as control [15]. No significant difference was observed in total intraoperative blood loss between TXA-treated group (1269±690 ml) and control group (1336±550 ml). A similar result was observed in a single-blind RCT of 40 patients undergoing cervical laminoplasty, in which TXA was administrated based on body weight (15 mg/kg body weight) and administered 15 minutes before the initial surgical incision [18]. The intraoperative blood loss in the TXA-treated group (49.1±30.6 ml) was not significantly different from that observed in the control group (63.4±53.0 ml; P = 0.30). Compared with the multiple doses administered in other studies showing positive results, the single administration of TXA in these two studies might contribute to the discrepancy. Further study will be required to examine the dosage-related effects in larger studies.

A recent meta-analysis has assessed the effect of TAX on blood transfusion, thromboembolic events, and mortality in surgical patients [29], reporting strong evidence of TAX in reducing bleeding and blood transfusion in patients undergoing thromboembolic events, such as myocardial infarction, stroke, DVT, and pulmonary embolism. However, the effect of TAX on thromboembolic events and mortality remains unclear. Ross et al. conducted a systematic review on the association of frequency of thrombotic events with TAX after spontaneous bleeding, finding the frequency of some events, such as DVT, decreased with TXA treatment [30], lower rate of several complication, such as DVT, pulmonary embolism (PE), and arterioocclusive events was observed when TXA was applied as a blood conservation modality during primary total hip and knee arthroplasty [31]. Although TXA has demonstrated to be effective in in mediating DVT in many types of surgery, the efficacy and safety of TXA in spinal surgery remains controversial. It was suggested in some studies, TXA may increase the risk of DVT and other thromboembolic events. Gulba et al. showed that TXA may actually reverse the effects of heparin, thus results in serious bleeding constantly in patients [32]. In our systematic review of perioperative IV TXA administration in spinal surgery, we did not observe any statistically significant increases thromboembolic event risk. However, due the small number of patients included and the number of events recorded, further larger studies are needed to confirm the observation.

Current study has been focused on the effects of perioperative administration of TXA independent of dosage and timing. Further exploration of intraoperative TXA administration is required to fully assess the potential benefits of widespread clinical administration. In one retrospective study, a 50% reduction in transfusion requirements were observed in patients treated with higher intraoperative doses of TXA [33]. However, higher TXA dosages are not necessarily effective in reducing blood loss and transfusion rates, and the mechanism remains largely unknown. Bednar et al. [34] found that a TXA infusion at a high dose (1 mg/kg/h) was ineffective in limiting blood loss during surgery in metastatic cancer of the spine. The subgroup analysis by dose regimen indicated similar results using either high or low dosages of TXA. However, we cannot exclude that significant heterogeneity in doses and administration timing in the included studies might contribute to the observation. Furthermore, differences in surgical techniques and transfusion thresholds may be contributing factors to performance bias.

In summary, perioperative IV TXA significantly reduces intra-operative blood loss and transfusion requirements for spinal surgery patients. Furthermore, TXA administration was not linked with any significant increase in complication rates, including DVT. Better post-operative outcomes are also achieved by the reduced total blood loss achieved by TXA administration. Considering the significant heterogeneity and relatively small number of studies included, further larger studies are needed to confirm the findings, as well as to determine the optimal TXA dosages and administration timing for spinal surgery patients.

## Supporting Information

Supporting Information S1 File containing the following tables Table S1. Characteristics of Included Randomized Controlled Trials and quasi-RCTs. Table S2. Subgroup analysis of outcome for low doses of perioperative IV TXA. Table S3. Subgroup analysis of outcomes for high doses of perioperative IV TXA. Table S4. Sensitivity analyses for excluded qi-RCTs.
(DOC)
