# Supplementary material for: Systematic Review and Meta-Analysis of Perioperative Intravenous Tranexamic Acid Use in Spinal Surgery
Source: PLoS One. 2013 Feb 12;8(2):e55436. doi: 10.1371/journal.pone.0055436 (PMC3570541; doi:10.1371/journal.pone.0055436)
Supplement: Supporting Information S1 File containing the following tables — Table S1. Characteristics of Included Randomized Controlled Trials and quasi-RCTs. Table S2. Subgroup analysis of outcome for low doses of perioperative IV TXA. Table S3. Subgroup analysis of outcomes for high doses of perioperative IV TXA. Table S4. Sensitivity analyses for excluded qi-RCTs. (DOC) [file pone.0055436.s001.doc]

Table S1.Characteristics of Included Randomized Controlled Trials and quasi-RCTs.

| **Author/s** | **Number**  **（TAX/Contral）** | **Design** | **Intervention** | **Anaesthesia** | **Surgical methods** | **DVT screen** | **Transfusion Criteria** |
| --- | --- | --- | --- | --- | --- | --- | --- |
| Neilipovitz et al（2001） | 22/18 | RCT | TAX:10 mg/kg and infusion of 1 mg/(kg · h)  Contral: saline | [general anesthesia](http://dict.cn/general anesthesia) | correction of scoliosis | Clinical | Hb＜70g/L |
| Sethna et al  （2005） | 23/21 | RCT | TAX:100 mg/kg and infusion of 10 mg/(kg · h)  Contral: saline | [general anesthesia](http://dict.cn/general anesthesia) | correction of scoliosis | Clinical | HCT＜25% |
| Wong et al  （2008） | 73/74 | RCT | TAX:10 mg/kg and infusion of 1 mg/(kg · h)  Contral: saline | [general anesthesia](http://dict.cn/general anesthesia) | Spinal Fusion Surgery | Clinical &  ultrasound | Hb＜70g/L |
| Elwatidy et al  （2008） | 32/32 | quasi-RCT | 2g(for adults) or 30 mg/kg (for children), and infusion of 100 mg/h (for adults) or 1 mg/kg/h (for children)  Contral: saline | [general anesthesia](http://dict.cn/general anesthesia) | Spine Surgery | Clinical | Hb＜90g/L&  HCT＜27% |
| Farrokhi  et al  （2011） | 38/38 | RCT | TAX:10 mg/kg and infusion of 1 mg/(kg · h)  Contral: saline | [general anesthesia](http://dict.cn/general anesthesia) | Spinal Fixation Surgery | Clinical | none |
| Kim et al  （2000） | 11/11 | RCT | TAX:15mg  Contral: saline | [general anesthesia](http://dict.cn/general anesthesia) | Spinal Surgery | Clinical &  ultrasound | none |
| Tsutsumimoto et al  （2011） | 20/20 | quasi-RCT | TAX:15 mg/kg  Contral: saline | [general anesthesia](http://dict.cn/general anesthesia) | Cervical Laminoplasty | Clinical | none |
| Huang et al  （2011） | 34/34 | RCT | TAX:10 mg/kg and infusion of 2 mg/(kg · h)  Contral: saline | [general anesthesia](http://dict.cn/general anesthesia) | Spinal Fixation Surgery | Clinical &  ultrasound | Hb＜90g/L |
| Jalaeian et al (2009)a | 20/20 | RCT | TAX:15 mg/kg and infusion of 1 mg/(kg · h)  Contral: saline | [general anesthesia](http://dict.cn/general anesthesia) | Lumbar Hernial Disc Resection | none | none |
| Jalaeian et al (2009)b | 20/20 | RCT | TAX:15 mg/kg and infusion of 1 mg/(kg · h)  Contral: saline | [general anesthesia](http://dict.cn/general anesthesia) | Lumbar Hernial Disc Resection | none | none |

Table S2.Subgroup analysis of outcome for low doses of perioperative IV TXA.

| Outcome | Studies | Participants | Favors | Mean Difference/RR/  OR [95% CI] | I2(%) | P Value |
| --- | --- | --- | --- | --- | --- | --- |
| Intra-operative Blood loss | 6 | 457 | TXA | -86.50 [-162.04, -10.97] | 56 | 0.02 |
| Post-operative Blood loss | 4 | 277 | TXA | -98.10 [-113.53, -82.66] | 58 | ＜0.00001 |
| Total Blood loss | 4 | 289 | TXA | -330.36 [-555.32, -105.39] | 84 | 0.004 |
| Blood transfusion rate | 5 | 353 | TXA | 0.66 [0.52, 0.83] | 0 | 0.004 |
| DVT | 5 | 371 | TXA | 0.34 [0.01, 8.16] | Not applicable | 0.50 |
| Transfused packed cells (mL) | 4 | 234 | TXA | -111.58 [-198.49, -24.68] | 53 | 0.01 |

Table S3.Subgroup analysis of outcomes for high doses of perioperative IV TXA

| Outcome | Studies | Participants | Favors | Mean Difference/RR/  OR [95% CI] | I^2^(%) | P Value |
| --- | --- | --- | --- | --- | --- | --- |
| Intra-operative Blood loss | 2 | 108 | TXA | -413.13 [-684.24, -142.02] | 69 | 0.003 |
| Post-operative Blood loss | 1 | 64 | TXA | -117.37 [-224.03, -10.71] | Not applicable | 0.03 |
| Total Blood loss | 2 | 108 | TXA | -582.63 [-1027.04, -138.22] | 43 | 0.01 |
| Blood transfusion rate | 2 | 108 | TXA | 0.63 [0.41, 0.96] | 71 | 0.03 |
| DVT | 2 | 108 |  | Not estimable | Not applicable | Not applicable |
| Transfused packed cells (mL) | 2 | 108 | TXA | -367.18 [-643.76, -90.60] | 0 | 0.009 |

table S4. Sensitivity analyses for excluded qi-RCTs

|  | RR | 95% CI | P Value |
| --- | --- | --- | --- |
| All the studies | 0.65 | [0.53, 0.80] | <0.0001 |
| RCT studies | 0.68 | [0.56, 0.84] | 0.003 |
